# Quality and Safety of Blood Products

**DOI:** 10.1155/2016/2482157

**Published:** 2016-12-29

**Authors:** Sandra Ramirez-Arcos, Denese C. Marks, Jason P. Acker, William P. Sheffield

**Affiliations:** ^1^Canadian Blood Services, Ottawa, ON, Canada K1G 4J5; ^2^Research and Development, Australian Red Cross Blood Service, Sydney, NSW, Australia; ^3^Canadian Blood Services, 8249-114 Street, Edmonton, AB, Canada T6G 2R8; ^4^Department of Laboratory Medicine and Pathology, University of Alberta, 8249-114 Street, Edmonton, AB, Canada T6G 2R8; ^5^Canadian Blood Services, Hamilton, ON, Canada; ^6^Department of Pathology and Molecular Medicine, McMaster University, HSC 4N66, 1200 Main Street West, Hamilton, ON, Canada L8N 3Z5

Blood components used for transfusion therapy in developed countries include platelet concentrates (PCs), red cell concentrates (RBCs), and plasma. Current Good Manufacturing Practices (cGMP) ensure that blood operators maintain the Safety, Quality, Identity, Potency, and Purity of blood components, criteria collectively encompassed by the acronym SQuIPP. As highlighted in the review article “Quality Assessment of Established and Emerging Blood Components for Transfusion” by J. P. Acker et al. from Canada and Australia, assuring that blood components maintain high quality during storage and benefit transfusion recipients is challenging due to the variable nature of biological raw materials, different manufacturing processes, and variation in quality testing methods. Adding to this complexity is the fact that some products are subjected to nontraditional manufacturing or storage conditions. The authors concluded that, despite the development of novel quality assays, current tests including coagulation factor VIII activity for plasma, pH and platelet yield for PCs, and hemolysis of RBCs are user-friendly and remain valuable tools to determine blood product quality. It is however recognized that controversy still persists among transfusion practitioners as to the link between in vitro quality parameters and posttransfusion clinical effectiveness of blood components.

E. Maurer-Spurej and K. Chipperfield in Canada in their article “Could Microparticles be the Universal Quality Indicator for Platelet Viability and Function?” addressed the issue of lack of relatedness between in vitro quality assessment and in vivo effectiveness specifically related to PCs. The authors cited donor variation as one of the major factors affecting PC quality and clinical efficacy and suggested that this property should be considered when targeting different patient groups. While homogeneous viable platelets may be ideal for prophylactic treatment of cancer patients, trauma patients may benefit more with transfusion of heterogeneous platelets. The authors proposed that routine PC screening should include a rapid noninvasive assay to determine microparticle content in PCs as a universal indicator of platelet quality.

Quality testing should ensure similarity in the SQuIPP criteria of blood components prepared by different manufacturing methods. A. W. Shih et al. in Canada in their article “Quantification of Cell-Free DNA in Red Blood Cell Units in Different Whole Blood Processing Methods” assessed the content of cell-free DNA during RBC storage. Cell-free DNA is released by neutrophils prior to leukocyte reduction, which occurs at different times during unit preparation by the two methods used in Canada, the RBC filtration method or the whole blood filtration method. In the RBC filtration process, whole blood units are stored at room temperature for up to 20 h before production of RBCs, plasma, and PCs, with leukocyte reduction occurring at room temperature. In the whole blood filtration method, whole blood is refrigerated within 8 hours of collection, leukoreduced in the cold, and stored before production of plasma and RBCs within 72 h of phlebotomy. The authors detected lower cell-free DNA content in RBC units prepared by the RBC filtration method compared to RBC units produced by the whole blood filtration method. The clinical significance of these findings is unknown and merits further investigation.

Variation of practice for quality testing of blood products exist not only between blood suppliers but also among related products. W. P. Sheffield et al. in Canada in their article “Stability of Thawed Apheresis Fresh-Frozen Plasma Stored for up to 120 Hours at 1°C to 6°C” have demonstrated that there is no reason to have divergent standards for storage of thawed plasma from apheresis or whole blood collections. Current Canadian standards mandate whole blood-derived plasma to be frozen within 24 h after blood collection, which can be thawed and stored for up to 5 days at 1–6°C. However, apheresis plasma should be frozen within 8 h after phlebotomy and can only be stored refrigerated for 24 h after thawing. The authors provided evidence that the activity of coagulation factors and fibrinogen was significantly higher in thawed apheresis plasma compared to whole blood-derived plasma throughout a 5-day storage period. Results of the study can therefore be used to propose an extension of the shelf-life of thawed apheresis plasma to 5 days in refrigeration, which would result in reduced wastage of this product in Canadian hospitals.

Safety of blood components considers the relative freedom from harmful effect to patients, directly or indirectly, of a prudently administered product taking into account the character of the product and the condition of the recipient at the time of the transfusion. To assure blood component safety, several measures are taken into consideration during product manufacturing and storage. Additionally, testing for infectious agents, including viruses, bacteria, and parasites, is routinely or seasonally performed by different blood operators. Variability in detection of infectious agents should be avoided to ensure valid surveillance data. A. C. Shrestha et al. from Australia in their article “A Comparative Study of Assay Performance of Commercial Hepatitis E Virus Enzyme-Linked Immunosorbent Assay Kits in Australian Blood Donor Samples” highlighted the poor agreement between the results obtained with two commercial enzyme-linked immunosorbent assays for the detection of IgM and IgG against hepatitis E virus in plasma samples. The study concluded that interpretation of serology results for hepatitis E virus should be done with caution.

Results of testing for the presence of infectious agents in blood components are one of the criteria used for donor deferral. K. Rooks et al. in Australia in their article “Mitigating the Risk of Transfusion-Transmitted Dengue in Australia” conducted a study to determine the prevalence of dengue virus RNA and nonstructural protein NS1 in plasma of donors living in high-risk areas during dengue outbreaks. Currently, donors residing in Australian dengue endemic areas are not allowed to donate fresh components and can only donate plasma for fractionation. The authors reported no detectable dengue RNA or antigen NS1 and no cases of dengue within blood donors. It was concluded that the risk of transfusion-transmitted dengue is likely low and implementing dengue detection is not necessary. Deferring high-risk donors is still the best practice to mitigate dengue transmission by transfusion in Australia.

Bacterial contamination of blood components, in particular PCs, continues to pose the highest transfusion infectious risk in industrialized countries. Measures implemented worldwide to mitigate that risk include donor screening, skin disinfection, first aliquot diversion, PC screening for the presence of bacteria, and pathogen inactivation technologies. M. Maclean et al. in the United Kingdom and the United States in their article “A New Proof of Concept in Bacterial Reduction: Antimicrobial Action of Violet-Blue Light (405 nm) in* Ex Vivo* Stored Plasma” described a method to inactivate bacteria in plasma samples based on the use of a 405 nm light emitting diode exposure system. This methodology does not require the use of photosensitizers as other currently used pathogen inactivation technologies. The authors showed 99% bacterial inactivation in low-volume plasma bags. Further validation is required to show system effectiveness in PCs, the blood component manifesting the highest risk for bacterial contamination.

Throughout this special issue, the authors described the challenges faced by blood product manufacturers to meet the highest quality and safety standards. Production of blood components is an evolving field and the development of new blood product containers, anticoagulants and additive solutions, and screening and treatment technologies require continuous optimization of procedures and consequent actualization of standards that govern the transfusion industry. Hopefully, the articles presented herein will stimulate the publication of other studies on current and underdevelopment practices related to improving the quality and safety of blood products for the benefit of transfusion patients.

*Sandra Ramirez-Arcos* *Denese C. Marks* *Jason P. Acker* *William P. Sheffield*

